# Comparative genome analysis among *Variovorax* species and genome guided aromatic compound degradation analysis emphasizing 4-hydroxybenzoate degradation in *Variovorax* sp. PAMC26660

**DOI:** 10.1186/s12864-022-08589-3

**Published:** 2022-05-18

**Authors:** Nisha Ghimire, Byeollee Kim, Chang-Muk Lee, Tae-Jin Oh

**Affiliations:** 1 Department of Life Science and Biochemical Engineering, Graduate School, SunMoon University, Asan, 31460 Korea; 2 Agricultural Microbiology Division, National Institute of Agricultural Sciences, Rural Development Administration, Jeonju, 55365 Korea; 3 Genome-based BioIT Convergence Institute, Asan, 31460 Korea; 4 Department of Pharmaceutical Engineering and Biotechnology, SunMoon University, Asan, 31460 South Korea

**Keywords:** *Variovorax* species, Genome, Pan-genome, Aromatic compound degradation, 4-hydroxybenzoate

## Abstract

**Background:**

While the genus *Variovorax* is known for its aromatic compound metabolism, no detailed study of the peripheral and central pathways of aromatic compound degradation has yet been reported. *Variovorax* sp. PAMC26660 is a lichen-associated bacterium isolated from Antarctica. The work presents the genome-based elucidation of peripheral and central catabolic pathways of aromatic compound degradation genes in *Variovorax* sp. PAMC26660. Additionally, the accessory, core and unique genes were identified among *Variovorax* species using the pan genome analysis tool. A detailed analysis of the genes related to xenobiotic metabolism revealed the potential roles of *Variovorax* sp. PAMC26660 and other species in bioremediation.

**Results:**

TYGS analysis, dDDH, phylogenetic placement and average nucleotide identity (ANI) analysis identified the strain as *Variovorax* sp. Cell morphology was assessed using scanning electron microscopy (SEM). On analysis of the core, accessory, and unique genes, xenobiotic metabolism accounted only for the accessory and unique genes. On detailed analysis of the aromatic compound catabolic genes, peripheral pathway related to 4-hydroxybenzoate (4-HB) degradation was found among all species while phenylacetate and tyrosine degradation pathways were present in most of the species including PAMC26660. Likewise, central catabolic pathways, like protocatechuate, gentisate, homogentisate, and phenylacetyl-CoA, were also present. The peripheral pathway for 4-HB degradation was functionally tested using PAMC26660, which resulted in the growth using it as a sole source of carbon.

**Conclusions:**

Computational tools for genome and pan genome analysis are important to understand the behavior of an organism. Xenobiotic metabolism-related genes, that only account for the accessory and unique genes infer evolution through events like lateral gene transfer, mutation and gene rearrangement. 4-HB, an aromatic compound present among lichen species is utilized by lichen-associated *Variovorax* sp. PAMC26660 as the sole source of carbon. The strain holds genes and pathways for its utilization. Overall, this study outlines the importance of *Variovorax* in bioremediation and presents the genomic information of the species.

**Supplementary Information:**

The online version contains supplementary material available at 10.1186/s12864-022-08589-3.

## Background

The bacterial genus *Variovorax* belongs to the phylum *Proteobacteria* and the *Comamonadaceae* family [1]. *Variovorax* sp. PAMC26660 in this study was isolated from lichen source obtained from Antarctica, whose complete genome has been deposited in the National Centre for Biotechnology Information (NCBI). Likewise, other *Variovorax* species have been isolated from diverse habitats (Table 1), like plant rhizosphere [2], glacier, and places contaminated with chemicals [3] and plastics (NCBI: biosample database), thus representing their abilities to adapt and survive in extreme environments. *Variovorax* species are also reported to contribute to xenobiotic biodegradation based on their ability to degrade aromatic compounds [4, 5]*.* However, less detailed study of the central and peripheral pathways of aromatic compound catabolism has been reported.

**Table 1:** General information about the isolation source of the complete genomes of *Variovorax* species submitted to the NCBI

Species name	GenBank Accession	Isolation source	Isolation country
*V.* sp. PAMC26660	CP060295.1	Lichen	Antarctica
*V.* sp. PAMC 28711	CP014517.1	Lichen	Antarctica
*V.* sp. PAMC28562	CP060296.1	Glacier	Uganda
*V.* sp. 38R	CP062121.1	Soil	France
*V.* sp. PBL-E5	LR594671.1	Linuron-contaminated soil	Denmark
*V.* sp. PBL-H6	LR594659.1	Linuron-contaminated soil	Belgium
*V.* sp. PBS-H4	LR594675.1	Linuron-contaminated soil	Belgium
*V.* sp. PDNC026	CP070343.1	Plastic debris in land/lake environment	USA
*V.* sp. PMC12	CP027773.1 and CP027774.1	Potting soil	South Korea: Wanju
*V.* sp. RA8	LR594662.1	Riverbed sediment	Japan
*V.* sp. RKNM96	CP046508.1	Soil	Canada
*V.* sp. SRS16	LR594666.1	Linuron-contaminated soil	Denmark
*V.* sp. WDL1	LR594689.1	Linuron-contaminated soil	Belgium
*V. paradoxus* 5C-2	CP045644.1	Soil	Russia
*V. paradoxus* VAI-C	CP063166.1	Collage campus turn soil	USA
*V. paradoxus* CSUSB	CP046622.1	Roots of *Helianthus annuus*	NA
*V. paradoxus* B4	CP003911.1 and CP003912.1	Polluted soil near a production plant of the chemical	NA
*V. paradoxus* EPS	CP002417.1	Sunflower rhizosphere community	USA
*V. paradoxus* S110	CP001635.1 and CP001636.1	Interior of the potato plant	NA
*V. boronicumulans* J1	CP023284.1	Soil	China

Microorganisms conserve and acquire pathways for aromatic compound catabolism. Aromatic compounds represent about 20% of the earth’s biomass [6]. They are common growth substrates for microorganisms, as well as significant environmental pollutants obtained from plant decomposition [7], petroleum [8], and anthropogenic activities [9]. Several peripheral and central catabolic pathways and degradation strategies for aromatic compound degradation are observed in microbial genomes [10–12] that can be used for bioremediation approaches to clean up aromatic contaminants from the environment. This study highlights the peripheral and central catabolic pathways for aromatic compound degradation among *Variovorax* species.

With the increasing number and advances in whole genome sequencing, the study of genomes and comparative genomics have become popular choices to understand the behavior of an organism [13]. Whole genome analysis and comparison can provide comprehensive information regarding metabolism, behavior to the changing environment, and bacterial adaptation to xenobiotic. Further, the pan genome analysis outlines the core, accessory, and unique genes among the strains of the same genus that can illustrate information regarding the variable genes and the conserved genes acquired with increasing generation [14]. Bacteria isolated from Antarctic regions can deliver important information of their adaptation to the habitat. We previously published CAZymes related study for the bacterial strains isolated from Antarctic lichen [15]. However, this study explores the bioremediation potential of *Variovorax* sp. PAMC26660 isolated from the Antarctic lichen, and performs genome comparison with other *Variovorax* species obtained from the NCBI. In this study, we applied different tools to analyze and compare the genomic features of all available whole-genome sequences of *Variovorax* species.

## Results and discussion

### TYGS analysis, phylogenetic relation, ANI analysis and morphology of *Variovorax* sp. PAMC26660

Determining the taxonomic position is crucial for classification, characterization and identification of bacteria. The genome of *Variovorax* sp. PAMC26660 was submitted to Type strain genome server (TYGS) for whole genome based taxonomic analysis, which identified the strain as a potential new species. TYGS compares the query genome with all type strain genomes (16,276) available in the TYGS database [16] where the intergenomic or intragenomic relations can be inferred through the auto-generated phylogeny and digital DNA-DNA hybridization (dDDH) values. The pairwise comparison between PAMC26660 and the closest type strains using dDDH is shown in Additional file 1: Table S1. The table contains dDDH values and confidence intervals for species and subspecies close to PAMC26660 using three different GGDC (Genome-to-Genome Distance calculator) formulas. The value did not match to the species and subspecies delineation thresholds of 70 and 79%, respectively. dDDH method considers all the complete and incomplete genomes for analysis so to alleviate the sequence length bias, d4 formula is immune to the problems caused by sequence length [17].

**Fig. 1:**
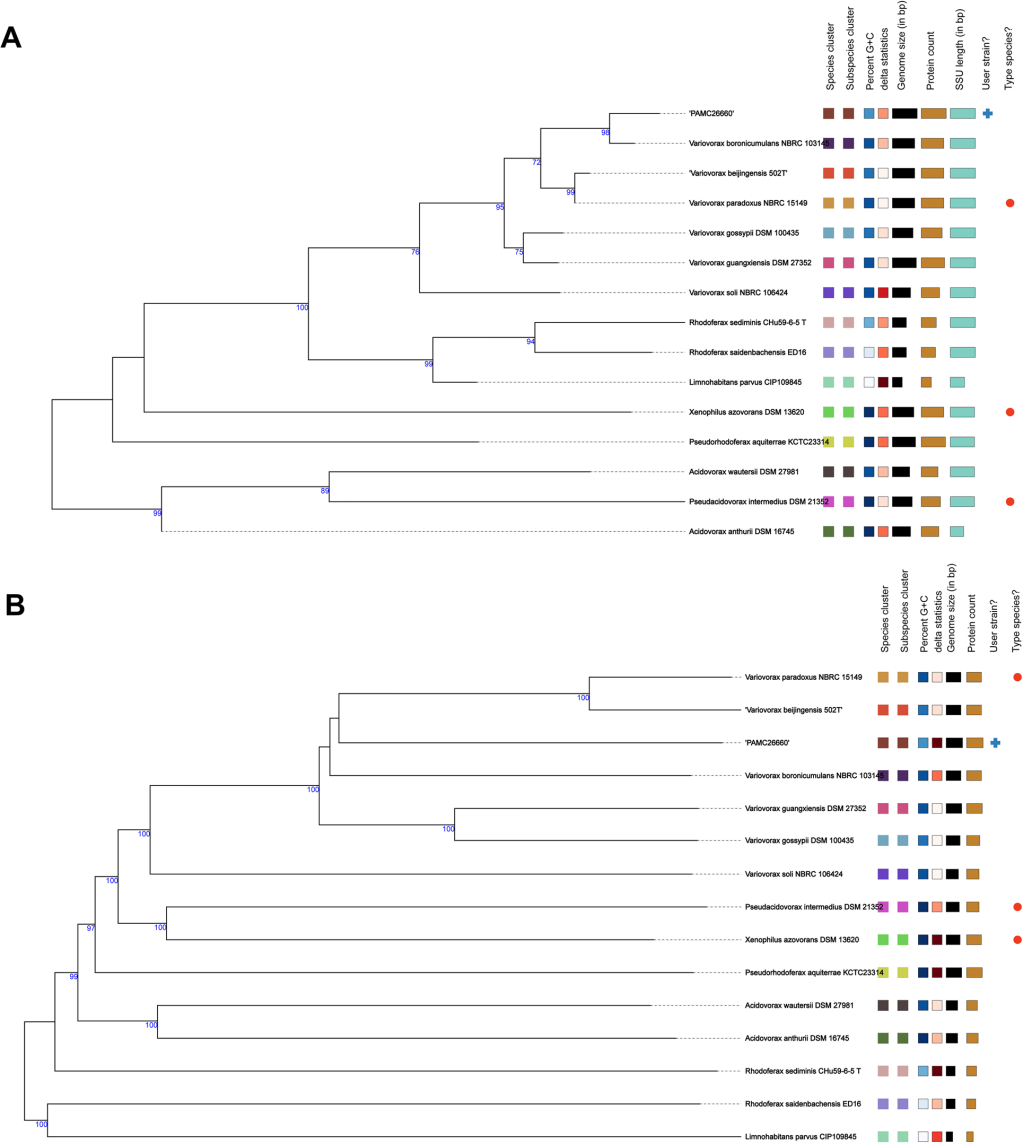
Genome BLAST Distance Phylogeny method (GBDP) for phylogenetic placement analysis using FastME 2.1.6.1. with 100 bootstrap values. (**A**) 16S rDNA gene sequence-based phylogeny of *Variovorax* sp. PAMC26660 with the closely related type strains and whole genomes with 87.4% average branch support. (**B**) Whole-genome sequence based phylogeny among the closely related type strains and whole genomes with 90.9% branch support. The numbers above branches represent the GBDP pseudo-bootstrap value, which is greater than 60%

The phylogenetic tree inferred from the intergenomic distance calculated from GBDP in the TYGS server is shown in Fig. 1. Based on the 16S rDNA comparison, PAMC26660 is closely related to *Variovorax boronicumulans* NBRC 103145, *Variovorax beijingensis* 502 T, and *Variovorax paradoxus* NBRC 15149, respectively, all clustered in the same clade (Fig. 1A). Similarly, the whole genome-based phylogeny also showed a cluster of the same species as the closest relatives of PAMC26660 (Fig. 1B). All the *Variovorax* species clustered together in a paraphyletic clade from the other type strains.

Besides, phylogenetic placement and dDDH, ANI analysis is another method for assessing evolutionary distance between bacterial species. Since the two other methods incorporated type strains along with complete and incomplete genomes, ANI analysis was performed only among the complete genomes of *Variovorax* species (characterized and uncharacterized species) to find the genomic relatedness among the same genus. In the ANI analysis performed using 3 different tools, the ANI values did not match the species delineation threshold i.e., (95 – 96) % identity to categorize PAMC26660 in any species (characterized or uncharacterized) of *Variovorax* (Table 2). However, PAMC26660 was closely related to uncharacterized species PDNC026 and PMC12 with maximum percentage identities obtained using all three tools. All the tools gave a reliable data with less variance. The OrthoANI is based on the identity between only the orthologous genes that are fragmented from the whole genome for analysis [18]. ANIb based on BLAST+ analysis within JspeciesWS is specific to the species-specific signatures [19] and FastANI is based on alignment-free approximate sequence mapping [20].

**Table 2:**
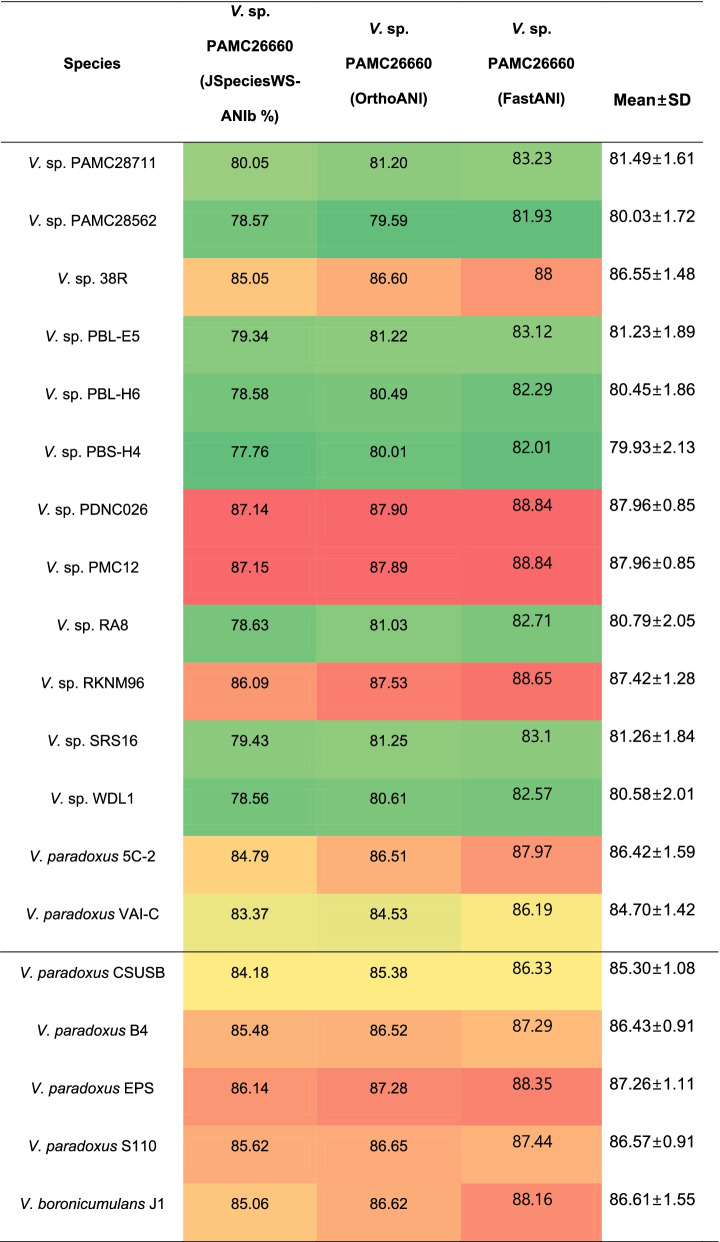
Average nucleotide identity based on ANIb, OrthoANI and FastANI

**Fig. 2:**
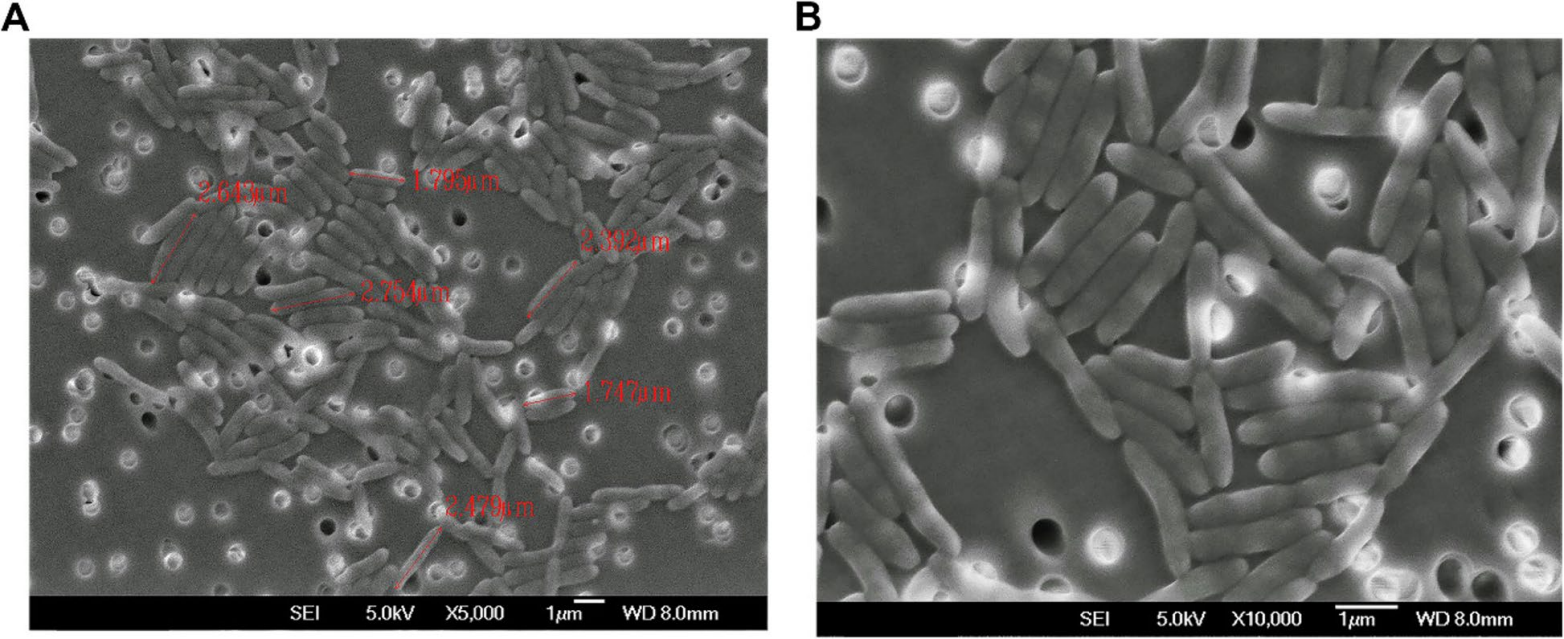
Morphology analysis of *Variovorax* sp. PAMC26660 as visualized by FE-SEM image shown at 5000× magnification (**A**), the image shown at 10,000× magnification (**B**)

Secondary electron images (SEI) of *Variovorax* sp. PAMC26660 were taken using Field Emission Scanning Electron Microscopy (FE-SEM) with 5 kV voltage, 8 mm working distance, and 1 μm scan width. Under the microscope, *Variovorax* sp. PAMC26660 appeared rod-shaped, straight to curved rods. They are clustered within groups in close proximity. Their length varied almost (1.7 – 2.8) μM (Fig. 2). According to Bergey’s manual, *Variovorax* species are gram negative, rod-shaped, with (0.5 – 0.6) μm diameter × (1.2 – 3.0) μm length, occurring in pairs or singly [21].

### Profile of *Variovorax* sp. PAMC26660 and functional annotation

*Variovorax* sp. PAMC26660 was isolated from the lichen in Antarctica whose complete genome has been submitted to NCBI with the accession number CP060295.1. PAMC26660 is composed of a single chromosome of 7,390,000 bp with 7023 protein coding genes (Additional file 1: Table S2). The coding genes were classified in different categories of RAST annotation server (Additional file 1: Fig. S1A). The most numerous categories in RAST annotation were amino acids and derivatives, carbohydrates and cofactors, vitamins, prosthetic groups, pigments, and protein metabolism, respectively. The metabolism of aromatic compounds category also accounted for quite a few genes. *Variovorax* species have been isolated from extreme environments and they have shown potential for bioremediation. Genome annotation of PAMC26660 from Antarctic lichen also contained genes for aromatic compound degradation, which has been explored in this study. Additionally, the RAST annotation data showed the strain to carry several stress-related genes that might hold responsible for survival in harsh environments like Antarctica. (Additional file 1: Table S3) indicates all the stress-related genes contained in the genome of PAMC26660. In the psychrophilic environment, microorganisms encounter stress conditions like osmotic pressure, excessive UV, low or high pH and low nutrient availability [22, 23]. Oxidative stress accounted for the highest stress response genes required to alleviate reactive oxygen species generated due to UV radiation followed by the osmotic stress, two of the prominent stress management strategies in a cold environment.

As per the interest of this study, the genes related to aromatic compound catabolism were further confirmed with KEGG annotation integrated into the JGI IMG database. Genome annotation with more than one tool can increase the confidentiality of the obtained data. KEGG annotation also accounted for quite a few genes related to aromatic compound catabolism. The 2161 protein coding genes connected to KEGG pathways were categorized into several KEGG categories as shown in (Additional file 1: Fig. S1B). The detailed study of the genes and pathways obtained from the annotation are explored in the following sections of this study.

### Core and pan genome analysis

Pan genome analysis accounts for the diversity among genomes by considering their core, accessory, and unique genes. Tettelin et al. proposed the pan genome to be the whole-genomic repertoire of a microorganism [24]. Pan genome analysis of all available genomes of *Variovorax* species resulted in the core, accessory, and unique genes. There are 103,717 accessory genes, 115 core genes, and 10,212 unique genes. The core genes referring to conserved genes were found to be very much less in number compared to the accessory and unique genes; this might refer to gene evolution through various events like mutation, gene rearrangement, or lateral gene transfer [25, 26]. Most of the genes that do not serve in the primary metabolic process are non-essential genes that evolve faster than the essential genes [27]. The non-essential genes account for the genes whose knock out does not affect the lethal phenotype and are prone to evolve faster. Xenobiotic metabolism does not serve in the primary metabolic processes in bacteria so the removal of these genes is not fatal to the organism. Most of the genes related to aromatic compounds catabolism in PAMC26660 might have evolved through mutation or gene rearrangement as only the homogentisate 1,2-dioxygenase gene was confirmed to be horizontally transferred by Island Viewer 4 tool [28] among the discussed aromatic compound catabolic pathways and genes in the current study. Island viewer 4 predicts the genomic islands through three different tools like IslandPath-DIMOB, SIGI-HMM, and IslandPick. Besides, 1511 genes in PAMC26660 were predicted to have undergone lateral gene transfer event whose detailed list has been provided in the Additional file 2: Table S4. The additional file 1: Fig. S2A represents the number of gene families among the genomes. The phylogenetic tree based on the core genes (Additional file 1: Fig. S2B) shows that PAMC26660 is closely related to PMC12 (chromosome 1) and PDNC026. The result was similar to the ANI analysis that also showed PMC12 and PDNC026 to be the closest relatives of PAMC26660.

### KEGG analysis

**Fig. 3:**
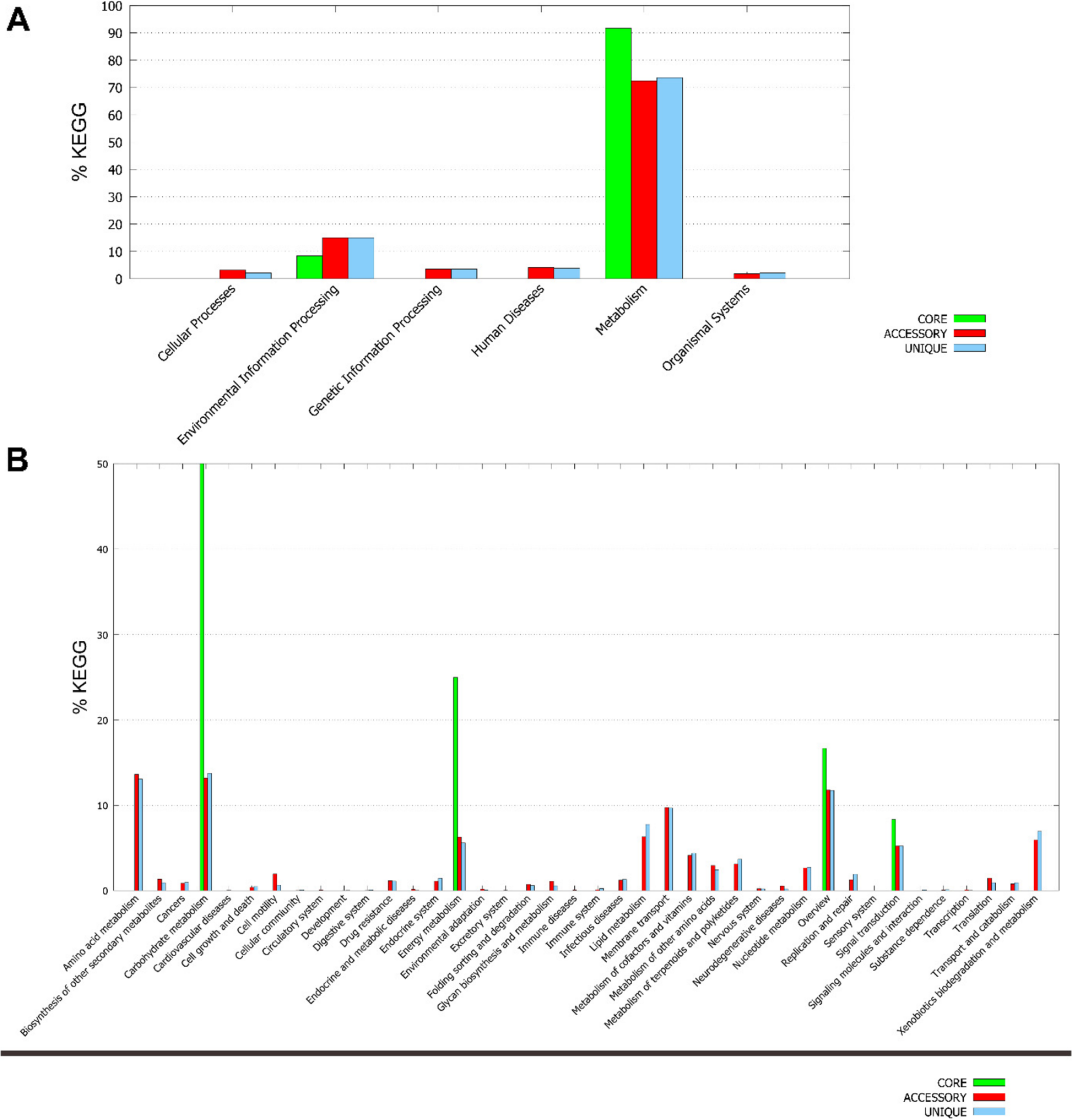
KEGG analysis of 20 *Variovorax* species using the core, accessory, and unique genes generated by pan genome analysis. (**A**) Represents distribution of the core, accessory, and unique genes among the KEGG main categories, while (**B**) represents the subcategories of the main categories in KEGG

The core, accessory, and unique genes obtained from the pan genome analysis were processed for functional analysis using the Kyoto Encyclopedia of Genes and Genomes (KEGG). This resulted in the distribution of these genes in several KEGG categories. The maximum number of genes accounted for metabolism, followed by environmental information processing, human disease, genetic information processing, cellular processes, and organismal system (Fig. 3A). Among the subcategories, carbohydrate metabolism accounted for the highest number of genes, followed by energy metabolism. The genes involved in carbohydrate and energy metabolism are functionally important genes compared to the other as molecular evolution theory postulates that the functionally important genes evolve slower [29, 30]. The xenobiotic metabolism also accounted for quite a few genes but as accessory and unique genes only (Fig. 3B) referring to the non-essential genes. However, not all bacteria can metabolize xenobiotic and these unique features hold environmental and biotechnological importance. On detailed analysis of the genes related to xenobiotic metabolism, we found peripheral pathways for aromatic compound catabolism, like 4-HB, tyrosine, and phenylacetate, and central catabolic pathways, like protocatechuate, phenylacetyl-CoA, and homogentisate catabolism that leads to the tricarboxylic acid (TCA) cycle in most of the *Variovorax* species studied (Additional file 3: Table S5).

### Central catabolic pathways for aromatic compound degradation in *Variovorax* sp. PAMC26660

**Fig. 4:**
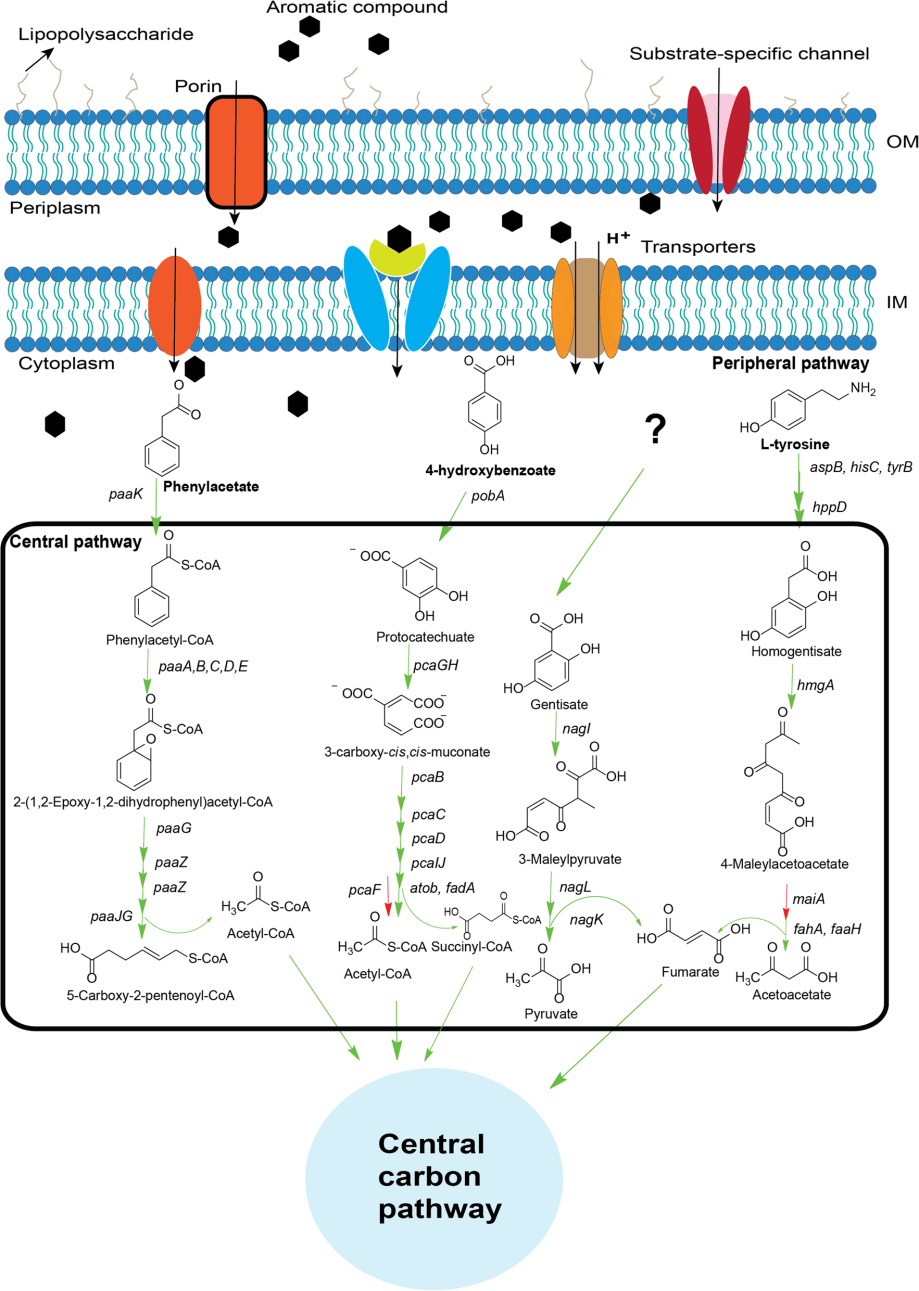
Proposed pathway for central and peripheral routes of the aromatic compound catabolism in *Variovorax* species obtained from KEGG pathway [41–43]. The green arrow symbolizes the presence, while the red symbolizes the absence of the gene in PAMC26660. Table S5 of the Additional file 3 shows the presence and absence of these genes for other *Variovorax* species

Central catabolic pathway intermediates are formed from the degradation of aromatic compounds belonging to the peripheral pathway of aromatic catabolism. On analysis of the genome of PAMC26660 in the xenobiotic metabolism category of the KEGG pathway database, the pathways related to the central route of aromatic compound degradation were revealed (Fig. 4). The intermediates of the central catabolic pathways include protocatechuate, catechol, homogentisate, gentisate, phenylacetyl-CoA, 3-(2,3-dihydroxyphenylpropionate), and 2,5-dihydroxynicotinate [31]. Among them, Table 3 shows the protocatechuate, homogentisate, gentisate, and phenylacetyl-CoA catabolic genes in PAMC26660. In the case of PAMC26660, the degradation of 4-HB results in the formation of protocatechuate, homogentisate from tyrosine and phenylacetyl-CoA from phenylacetate degradation based on the pathway map obtained from genomic analysis. But, the precursors for gentisate are yet to be discovered (Fig. 4). Our future study will delve into experimentally proving the roles of these central catabolic pathways for degrading peripheral pathway intermediates. Studies show that, homogentisate is the route for the aerobic catabolism of L-tyrosine, L-phenylalanine, and 3- and 4-hydroxyphenylacetate [32–34]. Similarly, *m*-hydroxybenzoate, 3-hydroxybenzoate, 2, 5-xylenol, and *m*-cresol degradation result in gentisate intermediate [35–37], while phenylalanine and styrene result in phenylacetyl-CoA intermediate [38] of central catabolism. These non-catechol hydroxyl-substituted aromatic carboxylic acids are the central intermediates formed from the upper pathways that begin with oxidation by either monooxygenase or dioxygenase in the aerobic condition [39, 40].

**Table 3:** Genomics-driven prediction of genes encoding peripheral and central catabolic pathways for aromatic compound degradation in *Variovorax* sp. PAMC26660

Gene	KEGG orthology ID	Locus Tag	Function	Reference gene/UniprotKB or PDB accession	Identity/query cover (%)
**4-hydroxybenzoate**					
*pobA*	K00481	5874025_5875206	4-hydroxybenzoate 3-monooxygenase	*pobA*/Q03298.1	67.26/99
**Protocatechuate**					
*pcaG*	K00448	1485975_1486583	protocatechuate 3,4-dioxygenase, alpha subunit	*pcxA*/P15109.1	61.46/94
*pcaH*	K00449	1485224_1485958	protocatechuate 3,4-dioxygenase, beta subunit	*pcxB*/P15110.1	73.04/94
*pcaB*	K01857	5194953_5196167	3-carboxy-cis,cis-muconate cycloisomerase	*pcaB*/P32427.3	45.79/88
*pcaC*	K01607	5196164_5196598	4-carboxymuconolactone decarboxylase	DC4C/P20370.2	34.92/87
*pcaD*	K01055	5197818_5198582	3-oxoadipate enol-lactonase	ELH2/P00632.3	37.71/92
*pcaI*	K01031	1440608_1441309	3-oxoadipate CoA-transferase, alpha subunit	*pcaI*/Q01103.2	70.70/92
*pcaJ*	K01032	1439964_1440611	3-oxoadipate CoA-transferase, beta subunit	*pcaJ*/P0A101.2	68.87/98
*atoB*	K00626	1438732_1439937	acetyl-CoA C-acetyltransferase	*pcaF*/Q43974.1	65.05/99
*fadA*	K00632	3392309_3393505 3439178_3440368 5029514_5030647	acetyl-CoA acyltransferase	*fadA*/O32177.1 *fadA*/O32177.1 *fadA*/Q010T4.1	50.64/98 45.45/99 50.77/99
**Tyrosine**					
*tyrB*	K00832	5638920_5640116	aromatic-amino-acid transaminase	*tyrB*/P04693.1	52.39/99
**Homogentisate**					
*hppD*	K00457	1745226_1746353	4-hydroxyphenylpyruvate dioxygenase	*hppD*/P80064.1	59.4/96
*hmgA*	K00451	2812778_2814088	homogentisate 1,2-dioxygenase	*hgd*/Q1D8L9.1	63.81/96
*faaH*	K16171	1772826_1773899	fumarylacetoacetate hydrolase	*faaH*/3LZK_A	58.26/92
*fahA*	K01555	2810513_2811778	fumarylacetoacetase	*faaA*/A5PKH3.1	47.12/94
**Gentisate**					
*nagI*	K00450	2892208_2893296	gentisate 1,2-dioxygenase	GDO1/Q9S3U6.1	52.91/95
*nagL*	K01801	5495938_5496576	maleylacetoacetate isomerase/maleylpyruvate isomerase	*nagL*/O86043.1	46.45/99
*nagK*	K16165	2893363_2894067	fumarylpyruvate hydrolase	*nagK*/O86042.1	48.19/81
**Phenylacetate**					
*paaK*	K01912	4002663_4003976	phenylacetate-CoA ligase	*paaK*/Q9L9C1.1	69.48/100
**Phenylacetyl-CoA**					
*paaA*	K02609	4001605_4002618	ring-1,2-phenylacetyl-CoA epoxidase subunit PaaA	*paaA*/P76077.1	65.16/91
*paaB*	K02610	4001306_4001608	ring-1,2-phenylacetyl-CoA epoxidase subunit PaaB	*paaB*/P76078.1	65.56/90
*paaC*	K02611	4000168_4000941	ring-1,2-phenylacetyl-CoA epoxidase subunit PaaC	*paaC*/P76079.1	49.79/94
*paaD*	K02612	3999644_4000168	ring-1,2-phenylacetyl-CoA epoxidase subunit PaaD	*paaD*/P76080.2	43.68/96
*paaE*	K02613	3998548_3999633	ring-1,2-phenylacetyl-CoA epoxidase subunit PaaE	*paaE*/P76081.1	42.34/99
*paaG*	K15866	4004803_4005606	2-(1,2-epoxy-1,2-dihydrophenyl) acetyl-CoA isomerase	*paaG*/P77467.1	55.13/98
*paaZ*	K02618	3991275_3993326	oxepin-CoA hydrolase / 3-oxo-5,6-dehydrosuberyl-CoA semialdehyde dehydrogenase	*paaZ*/P77455.1	58.74/99

### Peripheral 4-HB catabolic pathway and its utilization as sole carbon source in *Variovorax* sp. PAMC26660

**Fig. 5:**
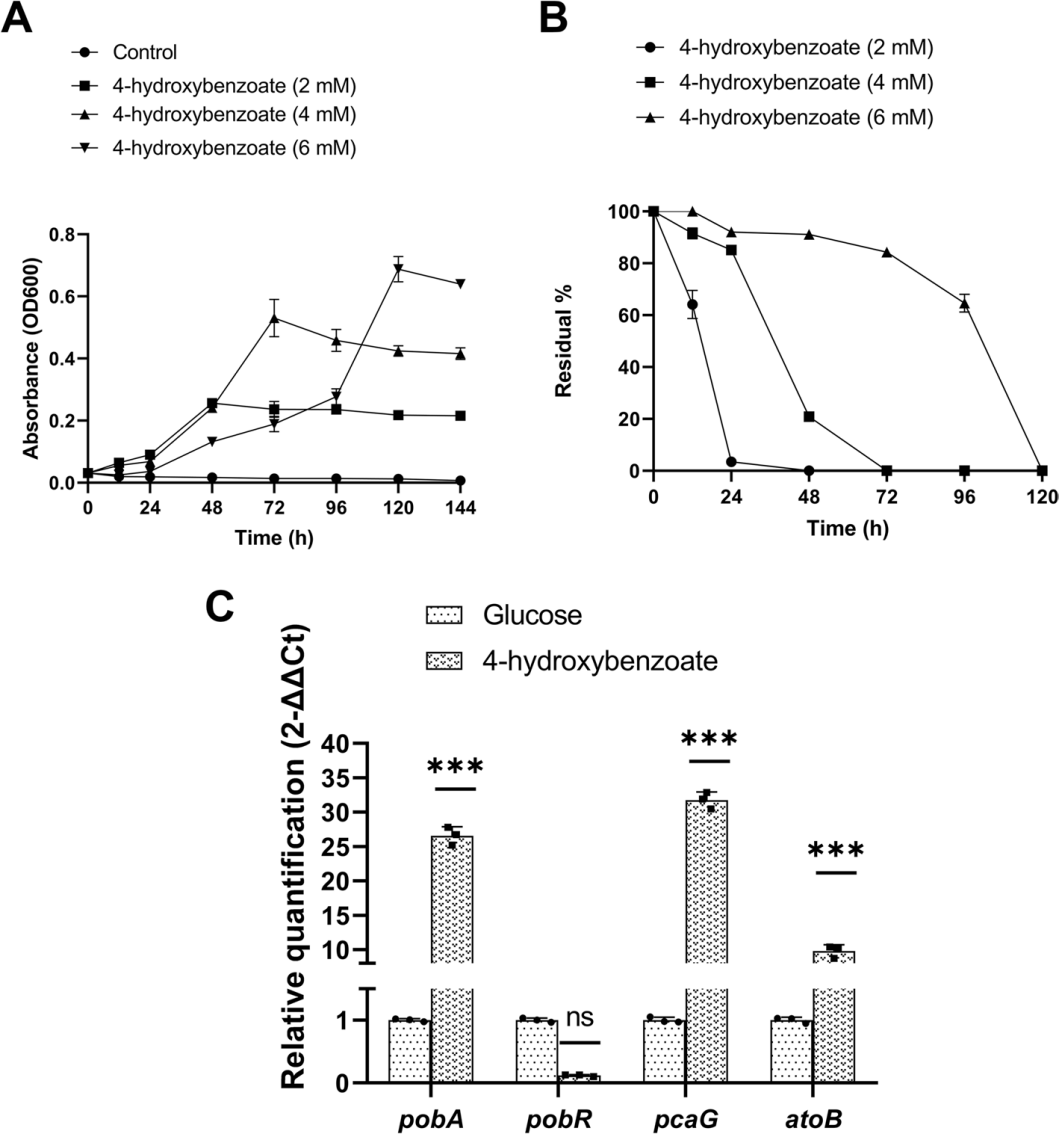
(**A**) Growth of *Variovorax* sp. PAMC26660 in the presence of 4-HB at different concentrations. (**B**) Residual percentage of 4-HB quantified by HPLC at different periods. (**C**) Relative quantification (fold change) of *pobA, pobR, pcaG*, and *atoB* genes of PAMC26660 strain in the presence of 4 mM of glucose (control) and 4-hydroxybenzoate (treated). Statistical analysis was performed using ANOVA test, followed by Bonferroni multiple comparison post hoc test with the statistically significant value of *p* < 0.05 (***P < 0.0001, ns; not significant)

4-HB is one of the aromatic compounds found in the lichen [44] (the isolation source of the strain), and its degradation was experimentally tested using *Variovorax* sp. PAMC26660. The genome of *Variovorax* sp. PAMC26660 contained peripheral pathway for 4-HB, tyrosine, and phenylacetate degradation that leads to protocatechuate, homogentisate, and phenylacetyl-CoA, respectively (Fig. 4). However, for this study, we tested the 4-HB degradation ability experimentally in PAMC26660 based on its isolation source. For this purpose, PAMC26660 was grown in 4-HB as a sole carbon source in mineral media, and the degradation of the compound was quantified using HPLC. Interestingly, in (2 and 4) mM 4-HB, PAMC26660 showed rapid growth, while in the case of 6 mM 4-HB, the growth was initially slow, which rapidly reached the stationary phase at 120 h (Fig. 5A). Based on the HPLC result (peak obtained at retention time: 7.5 min and 245 nm UV), 4-HB was completely absent in 1 mL aliquot within (24, 72, and 120) h for (2, 4, and 6) mM, respectively (Fig. 5B). The experimental result corresponds to the functionality of the genomic result obtained for the genes of 4-HB degradation. The growth inhibition of PAMC26660 with respect to increasing concentration of 4-HB was analyzed using 5–500 mM of 4-HB in mineral media as described earlier, the growth was slightly halted in 25 mM concentration while there was no growth from 50 to 500 mM concentration of 4-HB (Additional file 1: Fig. S3).

**Fig. 6:**
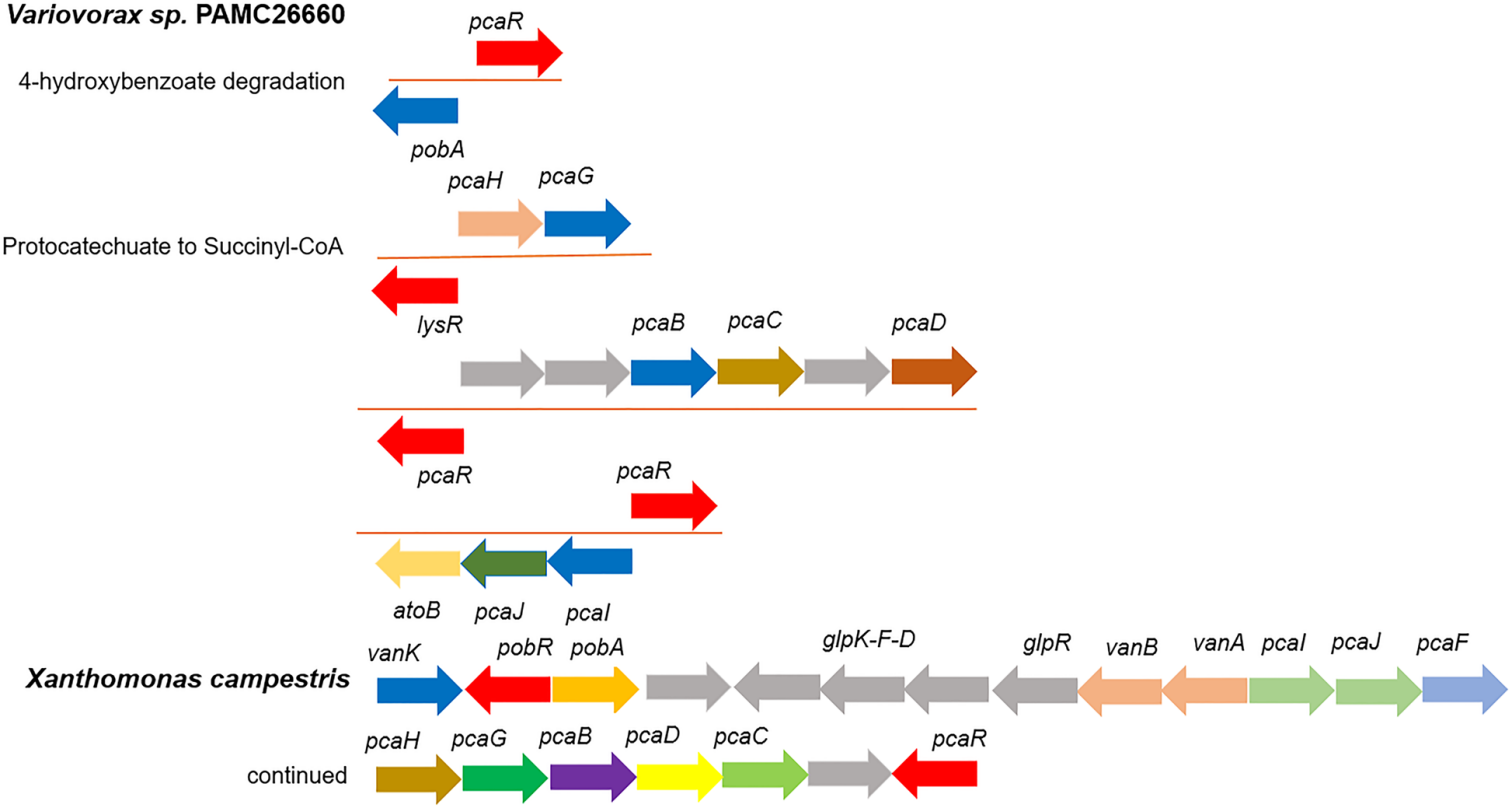
Distribution of the 4-HB degradation gene in an operon. The forward arrows indicate the genes in the positive strand, while the backward arrows indicate the genes in the negative strand. Grey arrows represent the genes that are not known to be involved in the catabolism process

Primarily, for the catabolism of 4-hydroxybenzoate membrane transporters are vital. RAST annotation data for the whole genome analysis of PAMC26660 showed two aromatic acid transporters specific to 4-hydroxybenzoate transport (Additional file 1: Table S3) under the category metabolism of aromatic compounds. Likewise, the AAHS family 4-hydroxybenzoate transporter-like MFS transporter (KO: K08195, 2894128_2895498) was detected in the KEGG annotation that showed 50.26% identity and 28% query cover with 4-hydroxybenzoate transporter from *Pseudomonas putida* (Q51955.1). The transported 4-HB is then hydroxylated to protocatechuate by 4HB-3-monooxygenase (*pobA*). Protocatechuate undergo oxygenolytic ring cleavage by PCA 3,4-dioxygenase (*pcaGH*) to form 3-carboxy-cis,cis-muconate, which is further converted by 3-carboxy-cis,cis-muconate cycloisomerase (*pcaB*) into 4-carboxymuconolactone. 4-carboxymuconolactone is converted into 3-oxoadipate-enol-lactone via 4-carboxymuconolactone decarboxylase (*pcaC*), and hydrolyzed to 3-oxoadipate by 3-oxoadipate enol-lactonase (*pcaD*). Further, 3-oxoadipate is converted into 3-oxoadipyl CoA, and finally to succinyl-CoA and acetyl-CoA by 3-oxoadipate CoA-transferase (*pcaIJ*) and acetyl-CoA C-acetyltransferase (*atoB*), respectively (Fig. 4). In *Burkholderia xenovorans* LB400, the protocatechuate degradation pathway consists of 3-oxoadipyl-CoA thiolase (*pcaF*) for the conversion of 3-oxoadipyl CoA to succinyl CoA and acetyl-CoA [45]. However, in the operon of PAMC26660*, pcaIJ,* and *atoB* were clustered together under the control of *pcaR* (Fig. 6 and Table 4). The *atoB* gene has 65.05% identity and 99% coverage with *pcaF*. We suppose the *atoB* gene lying in the same cluster, plays the role of *pcaF*, as it has good percentage identity with it. Also, while comparing to RAST annotation data, all the genes related to 4-HB catabolism were similar to KEGG annotation except for *atoB* (Additional file 1: Table S3). However, the relative gene expression of three key catabolic genes, *pobA*, *pcaG*, and *atoB*, from the 4-HB degradation pathway compared with the control sample (glucose) and the treated sample (4-HB) showed an increase in expression of *the atoB* gene. The overall result showed that the expression of all three genes increased in the treated sample (Fig. 5C) thus representing the protocatechuate mediated central catabolism of 4-HB that is converted to protocatechuate by the *pobA* gene, while *atoB* gene might function for the catabolism of 3-oxoadipyl CoA, as shown in Fig.4 which requires further experimental verification.

**Table 4:** Transcriptional regulators of the 4-HB degradation pathway through the central intermediate protocatechuate

Gene	Category from Fig. 6	Family	Reference gene/UniprotKB or PDB accession	Identity/query cover (%)	KEGG orthology ID	Locus Tag
*pcaR*	4-hydroxybenzoate	IclR	*ppobR*/Q43992.1	50.59/99	K02624	5875337_5876107
*lysR*	Protocatechuate to succinyl-coA	LysR	*PpcaQ*/P0A4T6.1	36.72/92	NA	1484130_1485119
*pcaR*	Protocatechuate to succinyl-coA	IclR	PcaU*PpcaU*/O83046.1	37.40/98	K02624	5,192,158–5,192,943
*pcaR*	Protocatechuate to succinyl-coA	IclR	*PpcaR*/Q52154.1	38.80/95	K02624	1,441,410–1,442,195

4-HB is an aromatic compound that is widely distributed in the plant kingdom [46]. They are low molecular weight lignocellulose derivatives formed during the degradation of lignin [47], which are eventually mineralized by soil microorganisms [48]. It was detected to exist in a free state in the soil [49]. Studies show that 4-HB falls under the category of phenolic acids, which is widely distributed among lichen species [44, 50]. Likewise, the degradation of some hydrocarbons, like toluene, cresol, and phenanthrene, have also been proposed to form 4-HB as intermediate of peripheral pathway [51, 52]. 4-HB degradation pathway in the phytopathogen, *Xanthomonas campestris* is claimed to contribute to full pathogenicity [53]. However, very much less information is available regarding the detailed mechanism of its occurrence. In comparison with other *Variovorax* species, all of them were found to include the complete pathway and genes for 4-HB degradation, while most of them contained, complete pathway for tyrosine and phenylacetate degradation (Additional file 3: Table S5).

### Genes involved in regulation of 4-HB degradation in *Variovorax* sp. PAMC26660

The regulation of 4-HB degradation occurs through transcriptional factors located in the operon containing the genes of the degradation pathway (Fig. 6 and Table 4). The regulation of aromatic compounds catabolic pathways is secondary to carbohydrates, therefore tight regulatory control occurs at the transcriptional level [54, 55]. The key enzyme for 4-HB degradation is *p*-hydroxybenzoate 3-monooxygenase (*pobA*). The common intermediate, protocatechuate, undergoes ring cleavage through protocatechuate 3, 4 dioxygenase alpha and beta subunit. PobR regulates the expression of *pobA*. It belongs to the ICIR family, and *pcaR* from PAMC26660 is close to *pobR* from *Acinetobacter calcoaceticus*, with 50.59% identity and 99% coverage [56]. A LysR family protein (36.72% identity and 92% coverage with *pcaQ*) was found in close proximity to *pcaG* and *pcaH*, key enzymes of protocatechuate degradation, while the other proteins of Pca regulon, *pcaB*, C, D, I, J, and *atoB* gene were under the control of *pcaR*. The regulatory and catabolic genes are not arranged in the same operon for 4-HB and protocatechuate in PAMC26660. However, *Xanthomonas campestris* possesses all the genes for the catabolism and regulation of 4-HB and protocatechuate clustered within the same operon [49] (Fig. 6). The relative gene expression analysis of *pcaR* or *pobR* located near the *pobA* gene of 4-HB degradation showed a decrease in expression fold change when grown in 4-HB containing media compared to glucose (Fig. 5C). Studies show that, PobR functions as both positive and negative regulator of *pobA* gene in the presence of 4-HB as an effector molecule. PobR from *Streptomyces coelicolor* was found to negatively regulate gene expression of *pobA* gene [57] while PobR from *Acinetobacter calcoaceticus* functioned as an activator [58]. The decrease in expression fold change of *pobR* depicts the role as a negative regulator; however, a gene knock out study is further required for the confirmation.

## Conclusions

In this study, we performed the genome and pan genome analysis of *Variovorax* sp. PAMC26660 with the complete genomes of *Variovorax*. *Variovorax* species contained numerous accessory and unique genes compared to core genes that might have evolved through mutation, gene recombination, or lateral gene transfer events. Through functional annotation using RAST and KEGG, *Variovorax* species carried genes and pathways for aromatic compound degradation. PAMC26660 consists of the 4-HB, tyrosine, and phenylacetate degrading peripheral pathways, while it consists of central catabolic pathways like, protocatechuate, homogentisate, gentisate, and phenylacetyl-coA of aromatic compound degradation. PAMC26660 could grow using 4-HB, an aromatic compound found in numerous lichen species as its sole carbon source. Its genome holds regulatory and catabolic genes for the biodegradation of 4-HB that can be used for metabolic engineering approaches or whole-cell biotransformation.

## Methods

### Isolation of *Variovorax* sp. PAMC26660, sequencing and annotation

The strain *Variovorax* sp. PAMC26660 was isolated from lichens from Antarctica obtained from the Korean Polar Research Institute (KOPRI, Incheon, Korea). It was isolated, sequenced, and annotated similarly to the way described in our previous paper for bacterial isolation from Antarctic lichen [15]. Genomic DNA was extracted from the single colony using QIAamp DNA Mini Kit (Qiagen Inc., Valencia, CA, USA). The purity of the strain was assessed by 16S rRNA sequencing amplified using two universal primers: 27F (5′- AGA GTT TGA TCM TGG CTC AG − 3′) and1492R (5′- GGT TAC CTT GTT ACG ACT T − 3′). The 16S rRNA gene sequence was compared with that in species strains available in the EzBioCloud database [59]. Genome sequencing was performed using PacBio RS II single-molecule real-time (SMRT) sequencing technology (Pacific Biosciences, Menlo Park, CA, USA) and the complete genome was submitted to NCBI. For genome annotation, the whole genome was submitted to rapid annotation subsystem technology (RAST) server [60] and the KEGG annotation was analyzed by the automated annotation under the JGI IMG database.

### TYGS analysis phylogenetic placement and ANI analysis

The whole genome sequence of PAMC26660 was uploaded to the Type Strain Genome Server (TYGS) for in silico based taxonomic analysis [16]. The pairwise comparison of the user strain with the type strains were performed using GBDP and accurate intergenomic distances inferred under the “trimming” algorithm and distance formula d5. Digital DDH values and confidence intervals were calculated following the recommended settings of GGDC 2.1 [16]. The intergenomic distances were used to create a balanced minimum evolution tree using FASTME 2.1.4 with 100 pseudo-bootstrap replicates for branch support [16]. ANI analysis was performed using three different methods like Orthologous Average Nucleotide Identity Software Tool (OAT) [18], JSpeciesWS [61] and FastANI [20].

### SEM analysis of *Variovorax* sp. PAMC26660 for morphology analysis

One mL of bacterial culture in tryptone soy broth (TSB) was centrifuged for 5 min at 13,000 rpm, and treated with 2.5% glutaraldehyde (500 μL) for 15 min. Cells were washed with 3D distilled water twice, and 100 μL of the culture was loaded in SEMPORE (JEOL). The cells on the surface were subjected to (40, 70, and 100) % ethanol, and air-dried. The prepared sample was platinum coated, and visualized using FE-SEM.

### Genome and pan genome analysis

For the genomic analysis, all the complete genomes (nucleotide and protein sequences) of 20 *Variovorax* species (available at the time of our study) and their genomes were downloaded from NCBI. Pan genome analysis was performed using all the complete genomes of *Variovorax* species employing the BPGA software package [62], using all the default parameters. Functional KEGG analysis and its distribution in core, pan, and accessory genome was performed using the advanced option in the BPGA software package. Further, pathway analysis was performed using KEGG [41], and joint genome institute (JGI) integrated microbial genomes (IMG) database [63] to find the genes related to aromatic compound degradation, and its regulatory proteins.

### Growth of *Variovorax* sp. PAMC26660 in 4-HB and utilization

*Variovorax* sp. PAMC26660 was grown in 4-HB as the sole carbon source. For this, 100 mM stock of sodium 4-HB (Tokyo Chemical Industry) was prepared in 3D water. To 250 ml flask containing 50 mL mineral media (0.2 g MgSO_4_, 0.02 g CaCl_2_, 1.0 g K_2_HPO_4_, 1.0 g KH_2_PO_4_, 1.0 g (NH_4_)_2_SO_4_, and 0.02 g FeSO_4_), (2, 4, and 6) mM of the compound was added. One mL of PAMC26660 grown on TSB (OD = 1, 25 °C) was added to each flask, and incubated at 25 °C. One mL aliquots of the culture were taken in different periods of (0, 12, 24, 48, 72, 96, 120, and 144) h, to analyze the growth of bacteria. All the experiments were performed in triplicate. Absorbance recorded at 600 nm using Biochrom Libra S35PC UV/visible spectrophotometer (Cambridge, UK) represented the turbidity and bacterial growth in the presence of 4-HB.

For the quantification of 4-HB in the culture, 1 mL of the culture was mixed with ethyl acetate (1:1), dried, and mixed with HPLC grade methanol. The sample was then filtered by 0.2 μm Whatman filter, and 20 μL was subjected to ultra-high performance liquid chromatography (U-HPLC, Thermo Fischer) instrument with Photodiode-Array Detector (PAD). The sample was separated using a Mightysil reverse-phase C18 column (4.6 mm × 250 mm, 5 μm; Kanto Chemical, Tokyo, Japan). Mobile phases acetonitrile (B) and water (A) were used in a gradient system of B at 10% for (0–1) min, 50% for (1–8) min, 70% for (8–14) min, 95% for (14–16) min, and 10% for (16–25) min, at a flow rate of 1 mL/min. Absorbance spectra of the substrates were monitored at 245 nm. The decrease in the area of the peak with respect to the control was analyzed at (0, 12, 24, 48, 72, 96, and 120) h to confirm substrate utilization by PAMC26660.

### Quantitative real-time PCR (qRT-PCR) for genes from the 4-HB catabolic pathway

Differential expression of 4 representative genes from the 4-HB catabolic pathway was analyzed using qRT–PCR (StepOnePlus™ Real-Time PCR System, Thermo Fisher Scientific) based on Comparative Ct (ΔΔCt) (relative quantitation) method. *Variovorax* sp. PAMC26660 was grown in mineral media containing 4 mM of glucose and 4-HB simultaneously as described above. The cells were harvested at mid log phase, centrifuged and RNA extraction was performed following the manufacturer’s protocol (PureLink™ RNA mini Kit, Invitrogen). cDNA synthesis was conducted using SuperScript™ VILO™ cDNA Synthesis Kit following manufacturer’s protocol. For qRT–PCR, the primers were designed using PrimerQuest tool (Additional file 4: Table S6). 16S rRNA was used as an endogenous control for normalization while using the comparative Ct method and glucose was kept as the control sample. SuperScript™ IV VILO™ Master Mix, Invitrogen containing SYBR green dye was used for qRT–PCR experiment. Each experiment was conducted in triplicate. The Ct values were compared for each gene by normalizing with 16S rRNA gene between the control samples (glucose) and treated samples (4-HB). Finally, the fold change or relative quantification was measured using 2^-ΔΔCt values.

## Supplementary Information

**Additional file 1: Supplementary Table S1.** Pairwise digital DNA-DNA hybridization values between query genome and the selected type strains and whole genomes by Type strain genome server. **Supplementary Table S2.** Genome features of *Variovorax* sp. PAMC26660. **Supplementary Figure S1.** (A) Bar graph representation of the number of genes assigned to each category in RAST annotation. (B) Bar graph representation of the number of genes assigned to each category in KEGG annotation. **Supplementary Table S3.** The stress related genes, aromatic compound catabolic genes and transporters existing in the genome of *Variovorax* sp. PAMC26660 based on RAST annotation. **Supplementary Figure S2.** Pan Genome analysis among all genomes of *Variovorax* species generated by the bacterial pan genome analysis (BPGA) pipeline. (A) Core and pan genome plot for the number of gene families among 20 *Variovorax* genomes. (B) Core phylogeny between *Variovorax* species that includes all the genes belonging to the core genome. **Supplementary Figure S3.** Growth inhibition of *Variovorax* sp. PAMC26660 grown in the presence of 4-HB at different concentrations.

**Additional file 2: Supplementary Table S4.** Laterally transferred genes predicted by Island Viewer 4 tool.

**Additional file 3: Supplementary Table S5.** Analyzing the presence (√) and absence (×) of genes related to peripheral and central aromatic compound catabolism pathway as shown by KEGG pathway analysis in each genome of *Variovorax* species.

**Additional file 4: Supplementary Table S6.** Primer designed for qRT–PCR.

## Data Availability

The datasets analyzed in the current study are available in the NCBI repository, accession numbers: CP060295.1 for *Variovorax* sp. PAMC26660, complete genome; CP014517.1 for *Variovorax* sp. PAMC 28711, complete genome; CP060296.1 for *Variovorax* sp. PAMC28562, complete genome; CP062121.1 for *Variovorax* sp. 38R, compete genome; LR594671.1 for *Variovorax* sp. PBL-E5, complete genome; LR594659.1 for *Variovorax* sp. PBL-H6, complete genome; LR594675.1 for *Variovorax* sp. PBS-H4, complete genome; CP070343.1 for *Variovorax* sp. PDNC026, complete genome; CP027773.1 and CP027774.1 for *Variovorax* sp. PMC12, complete genome; LR594662.1 for *Variovorax* sp. RA8, complete genome; CP046508.1 for *Variovorax* sp. RKNM96, complete genome; LR594666.1 for *Variovorax* sp. SRS16, complete genome; LR594689.1 for *Variovorax* sp. WDL1, complete genome; CP045644.1 for *Variovorax paradoxus* 5C-2, complete genome; CP063166.1 for *Variovorax paradoxus* VAI-C, complete genome; CP046622.1 for *Variovorax paradoxus* CSUSB, complete genome; CP003911.1 and CP003912.1 for *Variovorax paradoxus* B4, complete genome; CP002417.1 for *Variovorax paradoxus* EPS, complete genome; CP001635.1 and CP001636.1 for *Variovorax paradoxus* S110, complete genome; CP023284.1 for *Variovorax boronicumulans* J1, complete genome.

## Abbreviations

ANI: Average Nucleotide Identity
SEM: Scanning Electron Microscopy
4-HB: 4-hydroxybenzoate
NCBI: National Center for Biotechnology Information
SEI: Secondary Electron Images
FE-SEM: Field Emission Scanning Electron Microscopy
iTOL: Interactive Tree of Life
BPGA: Bacterial Pan Genome Analysis
KEGG: Kyoto Encyclopedia of Genes and Genomes
TCA: Tricarboxylic Acid
OAT: Orthologous Average Nucleotide Identity Software Tool
TSB: Tryptone Soy Broth
JGI: Joint Genome Institute
IMG: Integrated Microbial Genomes
PAD: Photodiode-Array Detector
