# Supplementary material for: Comparative genome analysis among Variovorax species and genome guided aromatic compound degradation analysis emphasizing 4-hydroxybenzoate degradation in Variovorax sp. PAMC26660
Source: BMC Genomics. 2022 May 18;23:375. doi: 10.1186/s12864-022-08589-3 (PMC9115942; doi:10.1186/s12864-022-08589-3)
Supplement: Supplementary file 1 — Additional file 1: Supplementary Table S1. Pairwise digital DNA-DNA hybridization values between query genome and the selected type strains and whole genomes by Type strain genome server. Supplementary Table S2. Genome features of Variovorax sp. PAMC26660. Supplementary Figure S1. (A) Bar graph representation of the number of genes assigned to each category in RAST annotation. (B) Bar graph representation of the number of genes assigned to each category in KEGG annotation. Supplementary Table S3. The stress related genes, aromatic compound catabolic genes and transporters existing in the genome of Variovorax sp. PAMC26660 based on RAST annotation. Supplementary Figure S2. Pan Genome analysis among all genomes of Variovorax species generated by the bacterial pan genome analysis (BPGA) pipeline. (A) Core and pan genome plot for the number of gene families among 20 Variovorax genomes. (B) Core phylogeny between Variovorax species that includes all the genes belonging to the core genome. Supplementary Figure S3. Growth inhibition of Variovorax sp. PAMC26660 grown in the presence of 4-HB at different concentrations. [file 12864_2022_8589_MOESM1_ESM.docx]

**Comparative genome analysis among *Variovorax* species and genome guided aromatic compound degradation analysis emphasizing 4-hydroxybenzoate degradation in *Variovorax* sp. PAMC26660**

Nisha Ghimire ^1^, Byeollee Kim^1^, Chang-Muk Lee^2^, and Tae-Jin Oh ^1,3,4*^

^1^ Department of Life Science and Biochemical Engineering, Graduate School, SunMoon University, Asan 31460, Korea

^2^ Agricultural Microbiology Division, National Institute of Agricultural Sciences, Rural Development Administration, Jeonju 55365, Korea

^3^ Genome-based BioIT Convergence Institute, Asan 31460, Korea

^4^ Department of Pharmaceutical Engineering and Biotechnology, SunMoon University, Asan 31460, Korea

^*^Corresponding authors: Prof. T.-J. Oh.

Department of Pharmaceutical Engineering and Biotechnology, SunMoon University, Asan 31460, South Korea. Tel.: +82 41 530 2677, E-mail: [tjoh3782@sunmoon.ac.kr](mailto:tjoh3782@sunmoon.ac.kr)

**Running title**: comparative genomics and aromatic compounds degradation by *Variovorax*

Table S1. Pairwise digital DNA-DNA hybridization values between query genome and the selected type strains and whole genomes by Type strain genome server

| **Query** | **Subject** | ***d_0_*** | **C.I. *d_0_*** | ***d_4_*** | **C.I. *d_4_*** | ***d_6_*** | **C.I. *d_6_*** |
| --- | --- | --- | --- | --- | --- | --- | --- |
| *Variovorax* sp. PAMC26660 | *Variovorax*  *boronicumulans* NBRC  103145 | 46.3 | [42.9 -  49.7] | 32.1 | [29.7 -  34.7] | 42.3 | [39.3 -  45.3] |
|  | *Variovorax gossypii* DSM  100435 | 39.9 | [36.5 -  43.4] | 31.6 | [29.2 -  34.1] | 37.1 | [34.2 -  40.2] |
|  | *Variovorax guangxiensis*  DSM 27352 | 47.5 | [44.1 -  50.9] | 31.5 | [29.1 -  34.0] | 43.0 | [40.0 -  46.0] |
|  | *Variovorax paradoxus*  NBRC 15149 | 37.6 | [34.2 -  41.1] | 31.4 | [29.0 -  33.9] | 35.3 | [32.3 -  38.3] |
|  | *Variovorax beijingensis*  502T | 37.2 | [33.8 -  40.7] | 31.2 | [28.8 -  33.7] | 34.9 | [31.9 -  38.0] |
|  | *Variovorax soli* NBRC  106424 | 24.1 | [20.8 -  27.8] | 22.8 | [20.5 -  25.3] | 22.8 | [20.0 -  25.9] |
|  | *Xenophilus azovorans*  DSM 13620 | 21.6 | [18.3 -  25.2] | 22.1 | [19.9 -  24.6] | 20.7 | [17.9 -  23.8] |
|  | *Pseudacidovorax*  *intermedius* DSM 21352 | 20.2 | [17.0 -  23.8] | 21.8 | [19.5 -  24.2] | 19.5 | [16.8 -  22.6] |
|  | *Acidovorax wautersii*  DSM 27981 | 17.3 | [14.2 -  20.8] | 21.5 | [19.2 -  23.9] | 17.1 | [14.5 -  20.1] |
|  | *Acidovorax anthurii* DSM  16745 | 16.3 | [13.4 -  19.8] | 21.2 | [19.0 -  23.6] | 16.3 | [13.7 -  19.2] |
|  | *Pseudorhodoferax*  *aquiterrae* KCTC23314 | 18.0 | [15.0 -  21.6] | 21.1 | [18.8 -  23.5] | 17.7 | [15.1 -  20.7] |
|  | *Rhodoferax sediminis*  CHu59-6-5 T | 16.7 | [13.7 -  20.2] | 20.8 | [18.6 -  23.2] | 16.6 | [14.0 -  19.5] |
|  | *Limnohabitans parvus*  CIP109845 | 13.9 | [11.1 -  17.3] | 19.5 | [17.3 -  21.9] | 14.1 | [11.7 -  17.0] |
|  | *Rhodoferax*  *saidenbachensis* ED16 | 14.5 | [11.7 -  17.9] | 19.4 | [17.2 -  21.8] | 14.7 | [12.2 -  17.5] |

confidence intervals (C.I.), Genome Blast Distance Phylogeny formulas (d_0_, d_4_ *and* d_6_)

Table S2. Genome features of *Variovorax* sp. PAMC26660

| **Feature** | **Value** |
| --- | --- |
| **Genome Statistics** |  |
| Total length bp | 7390000 bp |
| Contigs | 1 |
| N50 | 7,388,698 |
| L50 | 1 |
| GC% | 66% |
| **Genome features** |  |
| Assembly level | Complete Genome |
| Protein-coding genes | 7023 |
| Pseudogenes | 57 |
| rRNA genes | 6 |
| tRNA genes | 52 |


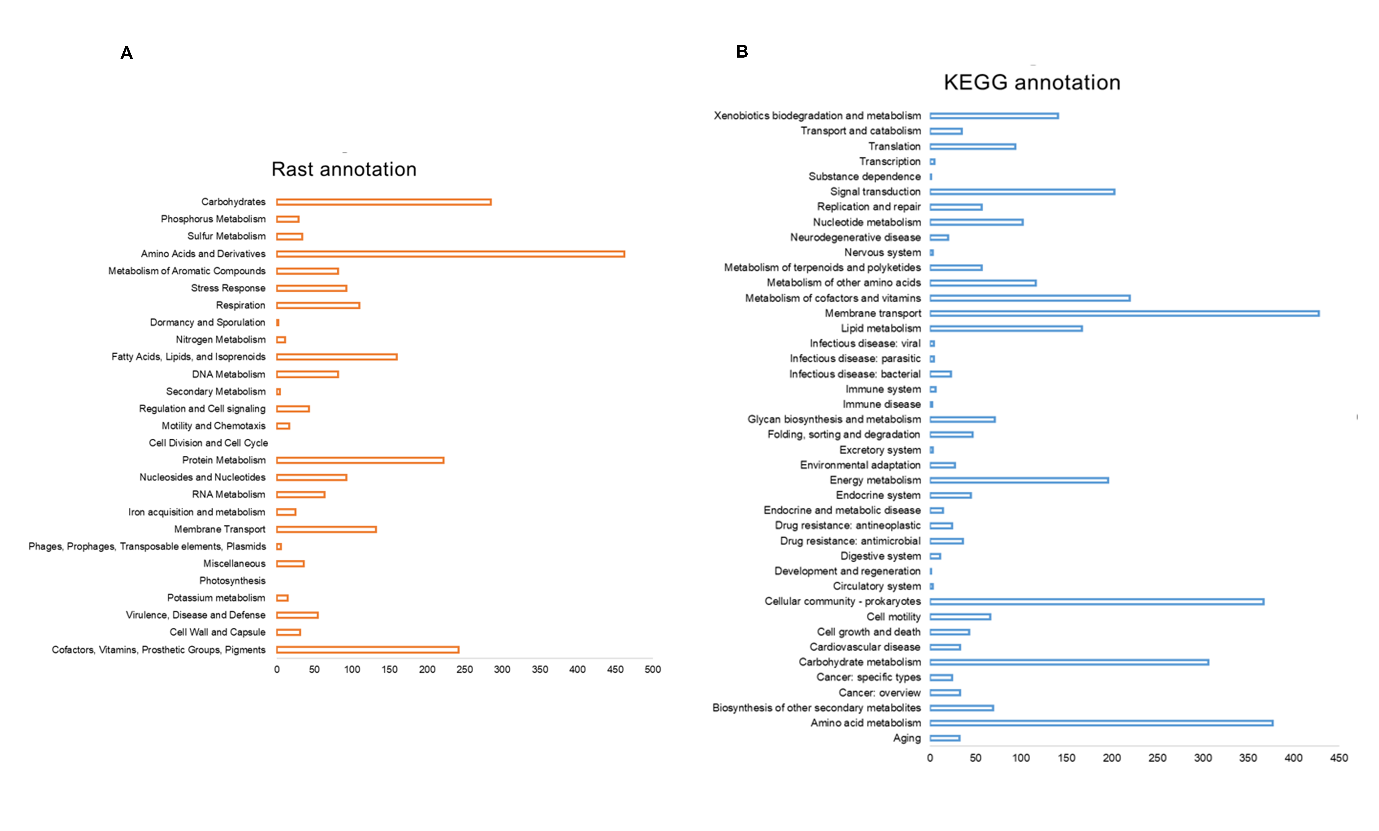


Fig. S1 (A) Bar graph representation of the number of genes assigned to each category in RAST annotation. (B) Bar graph representation of the number of genes assigned to each category in KEGG annotation.

Table S3. The stress related genes, aromatic compound catabolic genes and transporters existing in the genome of *Variovorax* sp. PAMC26660 based on RAST annotation

| **Stress response genes** | | | | |
| --- | --- | --- | --- | --- |
| Stress Response | Osmotic stress | Osmoregulation | Outer membrane protein A precursor | fig\|2762322.3.peg.4323, fig\|2762322.3.peg.5959, fig\|2762322.3.peg.5965 |
| Stress Response | Osmotic stress | Osmoregulation | Aquaporin Z | fig\|2762322.3.peg.1977 |
| Stress Response | Osmotic stress | Synthesis of osmoregulated periplasmic glucans | Cyclic beta-1,2-glucan synthase (EC 2.4.1.-) | fig\|2762322.3.peg.4037 |
| Stress Response | Osmotic stress | Synthesis of osmoregulated periplasmic glucans | OpgC protein | fig\|2762322.3.peg.4008 |
| Stress Response | Osmotic stress | Synthesis of osmoregulated periplasmic glucans | Glucans biosynthesis protein D precursor | fig\|2762322.3.peg.5045 |
| Stress Response | Osmotic stress | Choline and Betaine Uptake and Betaine Biosynthesis | Glycine betaine transporter OpuD | fig\|2762322.3.peg.2704 |
| Stress Response | Oxidative stress | Protection from Reactive Oxygen Species | Superoxide dismutase [Cu-Zn] precursor (EC 1.15.1.1) | fig\|2762322.3.peg.318 |
| Stress Response | Oxidative stress | Protection from Reactive Oxygen Species | Superoxide dismutase [Fe] (EC 1.15.1.1) | fig\|2762322.3.peg.2020, fig\|2762322.3.peg.6568 |
| Stress Response | Oxidative stress | Oxidative stress | Organic hydroperoxide resistance transcriptional regulator | fig\|2762322.3.peg.1944 |
| Stress Response | Oxidative stress | Oxidative stress | Transcriptional regulator, Crp/Fnr family | fig\|2762322.3.peg.2027, fig\|2762322.3.peg.2503, fig\|2762322.3.peg.3896, fig\|2762322.3.peg.6584, fig\|2762322.3.peg.6848 |
| Stress Response | Oxidative stress | Oxidative stress | Alkyl hydroperoxide reductase subunit C-like protein | fig\|2762322.3.peg.5584, fig\|2762322.3.peg.6206 |
| Stress Response | Oxidative stress | Oxidative stress | Fe2+/Zn2+ uptake regulation proteins | fig\|2762322.3.peg.171 |
| Stress Response | Oxidative stress | Oxidative stress | Superoxide dismutase [Cu-Zn] precursor (EC 1.15.1.1) | fig\|2762322.3.peg.318 |
| Stress Response | Oxidative stress | Oxidative stress | Paraquat-inducible protein B | fig\|2762322.3.peg.6061 |
| Stress Response | Oxidative stress | Oxidative stress | Superoxide dismutase [Fe] (EC 1.15.1.1) | fig\|2762322.3.peg.2020, fig\|2762322.3.peg.6568 |
| Stress Response | Oxidative stress | Oxidative stress | Peroxide stress regulator | fig\|2762322.3.peg.171 |
| Stress Response | Oxidative stress | Oxidative stress | Ferric uptake regulation protein FUR | fig\|2762322.3.peg.2973, fig\|2762322.3.peg.6776 |
| Stress Response | Oxidative stress | Oxidative stress | Superoxide dismutase [Mn] (EC 1.15.1.1) | fig\|2762322.3.peg.5581 |
| Stress Response | Oxidative stress | Oxidative stress | Ferric uptake regulation protein | fig\|2762322.3.peg.171 |
| Stress Response | Oxidative stress | Oxidative stress | transcriptional regulator, Crp/Fnr family | fig\|2762322.3.peg.1563, fig\|2762322.3.peg.6933 |
| Stress Response | Oxidative stress | Oxidative stress | Paraquat-inducible protein A | fig\|2762322.3.peg.6062, fig\|2762322.3.peg.6063 |
| Stress Response | Oxidative stress | Oxidative stress | Organic hydroperoxide resistance protein | fig\|2762322.3.peg.1945, fig\|2762322.3.peg.5189 |
| Stress Response | Oxidative stress | Glutathione: Biosynthesis and gamma-glutamyl cycle | Glutamate--cysteine ligase (EC 6.3.2.2), divergent, of Alpha- and Beta-proteobacteria type | fig\|2762322.3.peg.2994 |
| Stress Response | Oxidative stress | Glutathione: Biosynthesis and gamma-glutamyl cycle | Gamma-glutamyltranspeptidase (EC 2.3.2.2) | fig\|2762322.3.peg.3864, fig\|2762322.3.peg.4451, fig\|2762322.3.peg.4658 |
| Stress Response | Oxidative stress | Glutathione: Biosynthesis and gamma-glutamyl cycle | Putative exported protein clustered with Gamma-glutamyltranspeptidase | fig\|2762322.3.peg.3863 |
| Stress Response | Oxidative stress | Glutathione: Biosynthesis and gamma-glutamyl cycle | Glutathione synthetase (EC 6.3.2.3) | fig\|2762322.3.peg.2997 |
| Stress Response | Oxidative stress | Glutathione: Biosynthesis and gamma-glutamyl cycle | Glutamate--cysteine ligase (EC 6.3.2.2) | fig\|2762322.3.peg.184 |
| Stress Response | Oxidative stress | Glutathione: Non-redox reactions | Glutathione S-transferase (EC 2.5.1.18) | fig\|2762322.3.peg.347, fig\|2762322.3.peg.1502, fig\|2762322.3.peg.2442, fig\|2762322.3.peg.3512, fig\|2762322.3.peg.4537, fig\|2762322.3.peg.5111, fig\|2762322.3.peg.5261, fig\|2762322.3.peg.5262, fig\|2762322.3.peg.5531 |
| Stress Response | Oxidative stress | Glutathione: Non-redox reactions | Uncharacterized glutathione S-transferase-like protein | fig\|2762322.3.peg.3210 |
| Stress Response | Oxidative stress | Glutathione: Non-redox reactions | Lactoylglutathione lyase (EC 4.4.1.5) | fig\|2762322.3.peg.298, fig\|2762322.3.peg.5322 |
| Stress Response | Oxidative stress | Glutathione: Non-redox reactions | FIG005121: SAM-dependent methyltransferase (EC 2.1.1.-) | fig\|2762322.3.peg.5574 |
| Stress Response | Oxidative stress | Glutathione: Non-redox reactions | Glutathione S-transferase, unnamed subgroup (EC 2.5.1.18) | fig\|2762322.3.peg.1937, fig\|2762322.3.peg.4573 |
| Stress Response | Oxidative stress | Glutathione: Non-redox reactions | Glutathione S-transferase, unnamed subgroup 2 (EC 2.5.1.18) | fig\|2762322.3.peg.5490 |
| Stress Response | Oxidative stress | Glutathione: Non-redox reactions | Glutathione S-transferase family protein | fig\|2762322.3.peg.2206, fig\|2762322.3.peg.2849 |
| Stress Response | Oxidative stress | Glutathione: Non-redox reactions | Uncharacterized GST-like protein yncG | fig\|2762322.3.peg.6976 |
| Stress Response | Oxidative stress | Glutathione: Non-redox reactions | Hydroxyacylglutathione hydrolase (EC 3.1.2.6) | fig\|2762322.3.peg.5573 |
| Stress Response | Oxidative stress | Glutathione: Non-redox reactions | Probable glutathione S-transferase (EC 2.5.1.18), YfcF homolog | fig\|2762322.3.peg.1638 |
| Stress Response | Oxidative stress | Rubrerythrin | Rubredoxin | fig\|2762322.3.peg.1493 |
| Stress Response | Oxidative stress | Rubrerythrin | Alkyl hydroperoxide reductase subunit C-like protein | fig\|2762322.3.peg.5584, fig\|2762322.3.peg.6206 |
| Stress Response | Oxidative stress | Glutathione: Redox cycle | Glutathione reductase (EC 1.8.1.7) | fig\|2762322.3.peg.1923 |
| Stress Response | Oxidative stress | Glutathione: Redox cycle | Glutathione peroxidase (EC 1.11.1.9) | fig\|2762322.3.peg.2200, fig\|2762322.3.peg.2679 |
| Stress Response | Oxidative stress | Glutathione: Redox cycle | Glutaredoxin 3 (Grx3) | fig\|2762322.3.peg.971 |
| Stress Response | Oxidative stress | Glutaredoxins | Glutaredoxin 3 (Grx3) | fig\|2762322.3.peg.971 |
| Stress Response | Oxidative stress | Glutaredoxins | Glutaredoxin-related protein | fig\|2762322.3.peg.1024 |
| Stress Response | Detoxification | Uptake of selenate and selenite | Sulfate and thiosulfate import ATP-binding protein CysA (EC 3.6.3.25) | fig\|2762322.3.peg.6201 |
| Stress Response | Detoxification | Uptake of selenate and selenite | DedA protein | fig\|2762322.3.peg.5963, fig\|2762322.3.peg.6982 |
| Stress Response | Detoxification | Glutathione-dependent pathway of formaldehyde detoxification | S-formylglutathione hydrolase (EC 3.1.2.12) | fig\|2762322.3.peg.4570 |
| Stress Response | Detoxification | Glutathione-dependent pathway of formaldehyde detoxification | S-(hydroxymethyl)glutathione dehydrogenase (EC 1.1.1.284) | fig\|2762322.3.peg.4572 |
| Stress Response | Stress Response - no subcategory | SigmaB stress response regulation | Anti-sigma B factor antagonist RsbV | fig\|2762322.3.peg.6387 |
| Stress Response | Stress Response - no subcategory | Bacterial hemoglobins | diguanylate cyclase/phosphodiesterase (GGDEF & EAL domains) with PAS/PAC sensor(s) | fig\|2762322.3.peg.691, fig\|2762322.3.peg.6125, fig\|2762322.3.peg.6174 |
| Stress Response | Stress Response - no subcategory | Bacterial hemoglobins | Hemoglobin-like protein HbO | fig\|2762322.3.peg.891 |
| Stress Response | Stress Response - no subcategory | Hfl operon | Putative inner membrane protein YjeT (clustered with HflC) | fig\|2762322.3.peg.5452 |
| Stress Response | Stress Response - no subcategory | Hfl operon | HflC protein | fig\|2762322.3.peg.5451 |
| Stress Response | Stress Response - no subcategory | Hfl operon | RNA-binding protein Hfq | fig\|2762322.3.peg.5448 |
| Stress Response | Stress Response - no subcategory | Hfl operon | HflK protein | fig\|2762322.3.peg.5450 |
| Stress Response | Stress Response - no subcategory | Carbon Starvation | Carbon starvation protein A | fig\|2762322.3.peg.705 |
| Stress Response | Stress Response - no subcategory | Carbon Starvation | Stringent starvation protein A | fig\|2762322.3.peg.3768 |
| Stress Response | Periplasmic Stress | Periplasmic Stress Response | Sigma factor RpoE negative regulatory protein RseA | fig\|2762322.3.peg.4064 |
| Stress Response | Periplasmic Stress | Periplasmic Stress Response | Outer membrane stress sensor protease DegS | fig\|2762322.3.peg.3757 |
| Stress Response | Periplasmic Stress | Periplasmic Stress Response | Outer membrane protein H precursor | fig\|2762322.3.peg.6605 |
| Stress Response | Periplasmic Stress | Periplasmic Stress Response | Sigma factor RpoE negative regulatory protein RseB precursor | fig\|2762322.3.peg.4065 |
| Stress Response | Periplasmic Stress | Periplasmic Stress Response | HtrA protease/chaperone protein | fig\|2762322.3.peg.4066 |
| **Metabolism of aromatic compound and transporters** | | | | |
| Metabolism of Aromatic Compounds | Metabolism of central aromatic intermediates | p-Hydroxybenzoate degradation | P-hydroxybenzoate hydroxylase (EC 1.14.13.2) | fig\|2762322.3.peg.5630 |
| Metabolism of Aromatic Compounds | Metabolism of central aromatic intermediates | Homogentisate pathway of aromatic compound degradation | Transcriptional regulator, IclR family | fig\|2762322.3.peg.125, fig\|2762322.3.peg.459, fig\|2762322.3.peg.462, fig\|2762322.3.peg.470, fig\|2762322.3.peg.659, fig\|2762322.3.peg.1480, fig\|2762322.3.peg.1633, fig\|2762322.3.peg.2235, fig\|2762322.3.peg.2623, fig\|2762322.3.peg.2757, fig\|2762322.3.peg.2778, fig\|2762322.3.peg.3020, fig\|2762322.3.peg.4974, fig\|2762322.3.peg.5378, fig\|2762322.3.peg.5631, fig\|2762322.3.peg.5882, fig\|2762322.3.peg.6901, fig\|2762322.3.peg.6954 |
| Metabolism of Aromatic Compounds | Metabolism of central aromatic intermediates | Protocatechuate branch of beta-ketoadipate pathway | 3-oxoadipate CoA-transferase subunit B (EC 2.8.3.6) | fig\|2762322.3.peg.1410 |
| Metabolism of Aromatic Compounds | Metabolism of central aromatic intermediates | Protocatechuate branch of beta-ketoadipate pathway | 3-oxoadipate CoA-transferase subunit A (EC 2.8.3.6) | fig\|2762322.3.peg.1411 |
| Metabolism of Aromatic Compounds | Metabolism of central aromatic intermediates | Protocatechuate branch of beta-ketoadipate pathway | dicarboxylic acid transporter PcaT | fig\|2762322.3.peg.642, fig\|2762322.3.peg.3957 |
| Metabolism of Aromatic Compounds | Metabolism of central aromatic intermediates | Protocatechuate branch of beta-ketoadipate pathway | 4-carboxymuconolactone decarboxylase (EC 4.1.1.44) | fig\|2762322.3.peg.274, fig\|2762322.3.peg.4978 |
| Metabolism of Aromatic Compounds | Metabolism of central aromatic intermediates | Protocatechuate branch of beta-ketoadipate pathway | Succinyl-CoA:3-ketoacid-coenzyme A transferase subunit B (EC 2.8.3.5) | fig\|2762322.3.peg.5083 |
| Metabolism of Aromatic Compounds | Metabolism of central aromatic intermediates | Protocatechuate branch of beta-ketoadipate pathway | Succinyl-CoA:3-ketoacid-coenzyme A transferase subunit A (EC 2.8.3.5) | fig\|2762322.3.peg.5082 |
| Metabolism of Aromatic Compounds | Metabolism of central aromatic intermediates | Protocatechuate branch of beta-ketoadipate pathway | Pca regulon regulatory protein PcaR | fig\|2762322.3.peg.1412 |
| Metabolism of Aromatic Compounds | Metabolism of central aromatic intermediates | Protocatechuate branch of beta-ketoadipate pathway | Beta-ketoadipate enol-lactone hydrolase (EC 3.1.1.24) | fig\|2762322.3.peg.4980 |
| Metabolism of Aromatic Compounds | Metabolism of central aromatic intermediates | Protocatechuate branch of beta-ketoadipate pathway | Protocatechuate 3,4-dioxygenase beta chain (EC 1.13.11.3) | fig\|2762322.3.peg.1448 |
| Metabolism of Aromatic Compounds | Metabolism of central aromatic intermediates | Protocatechuate branch of beta-ketoadipate pathway | 3-carboxy-cis,cis-muconate cycloisomerase (EC 5.5.1.2) | fig\|2762322.3.peg.4977 |
| Metabolism of Aromatic Compounds | Metabolism of central aromatic intermediates | Protocatechuate branch of beta-ketoadipate pathway | Protocatechuate 3,4-dioxygenase alpha chain (EC 1.13.11.3) | fig\|2762322.3.peg.1449 |
| Metabolism of Aromatic Compounds | Peripheral pathways for catabolism of aromatic compounds | p-Hydroxybenzoate degradation | 4-hydroxybenzoate transporter | fig\|2762322.3.peg.1381 |
| Metabolism of Aromatic Compounds | Metabolism of central aromatic intermediates | Salicylate and gentisate catabolism | 4-hydroxybenzoate transporter | fig\|2762322.3.peg.1381 |


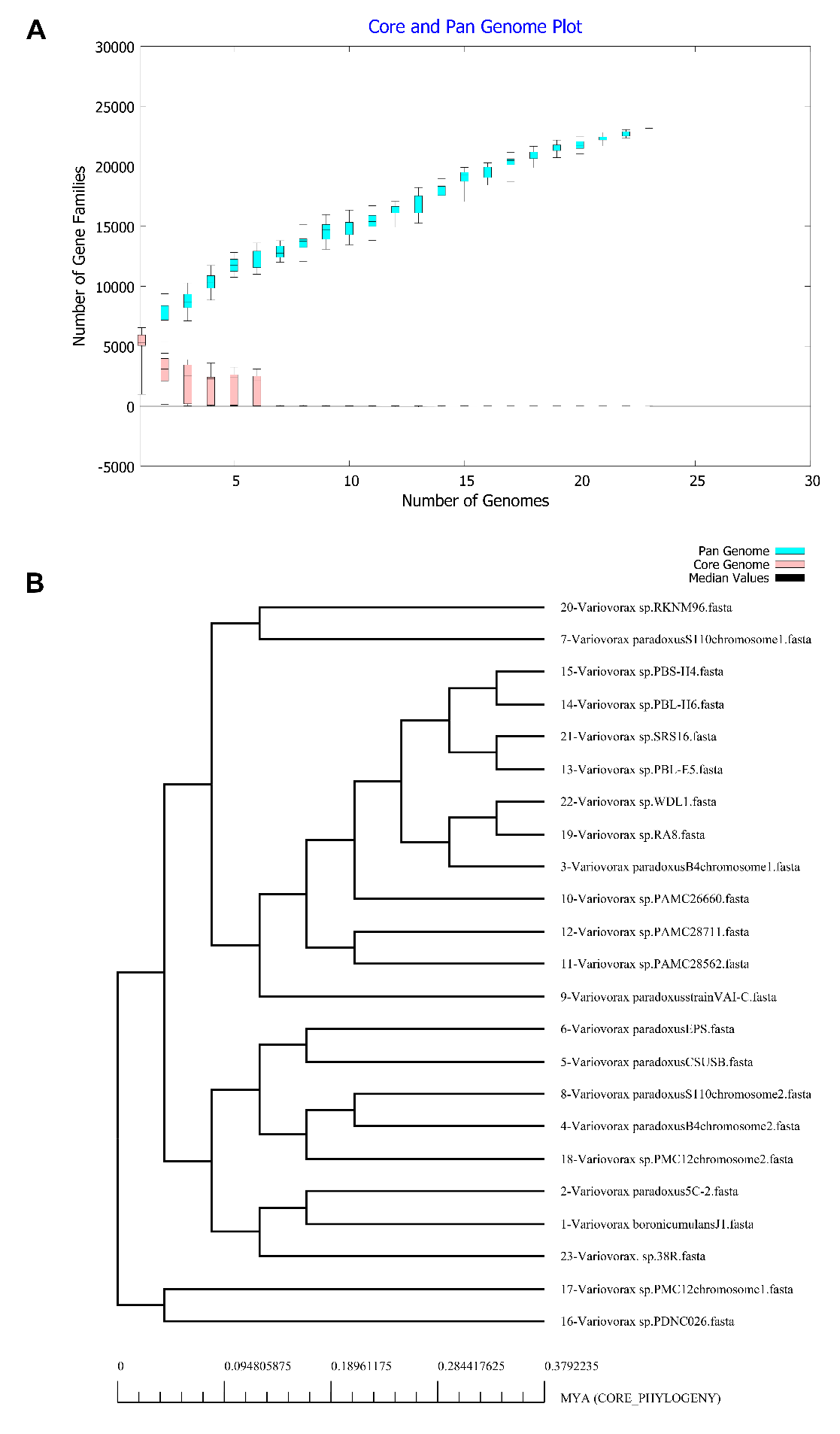


Fig. S2 Pan Genome analysis among all the chromosomes of *Variovorax* species generated by the bacterial pan genome analysis (BPGA) pipeline. (A) Core and pan genome plot for the number of gene families among 20 *Variovorax* genomes. (B) Core phylogeny between *Variovorax* species that includes all the genes belonging to the core genome.


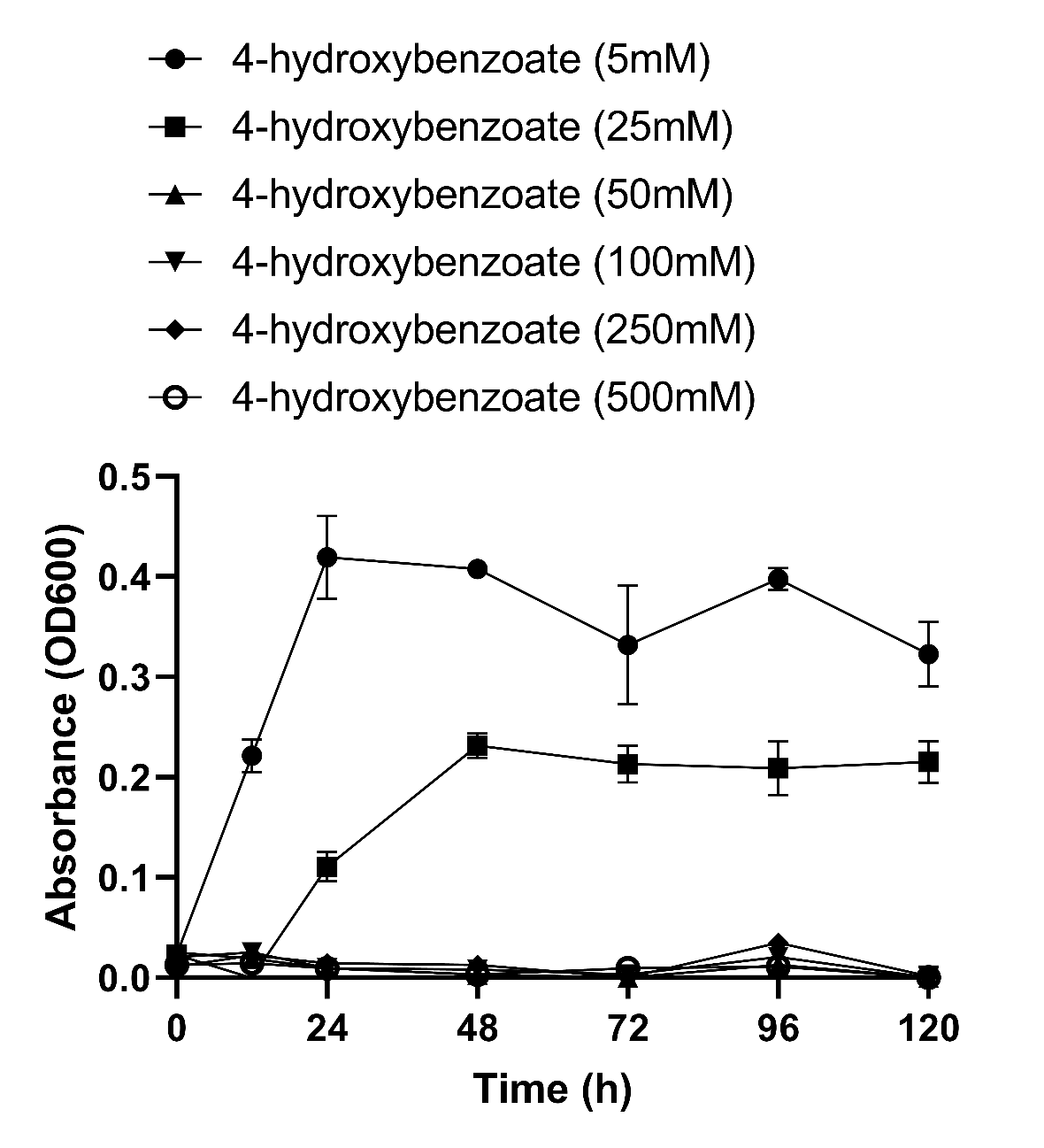


Fig. S3 Growth inhibition of *Variovorax* sp. PAMC26660 grown in the presence of 4-HB at different concentration.
